# Primary spinal anaplastic ependymoma: A single-institute retrospective cohort and systematic review

**DOI:** 10.3389/fonc.2023.1083085

**Published:** 2023-02-07

**Authors:** Liang Wu, Li’ao Wang, Wanjing Zou, Jun Yang, Wenqing Jia, Yulun Xu

**Affiliations:** ^1^ Department of Neurosurgery, Beijing Tiantan Hospital, Capital Medical University, Beijing, China; ^2^ Department of Pathology, Beijing Tiantan Hospital, Capital Medical University, Beijing, China

**Keywords:** primary spinal anaplastic ependymoma, clinical characteristics, treatments, progression-free survival, prognostic factors

## Abstract

**Objective:**

Primary spinal anaplastic ependymoma (PSAE) is an extremely rare disease. We aim to report the largest PSAE cohort, evaluate the treatments, and investigate the prognostic factors for progression-free survival (PFS).

**Methods:**

Clinical data collected from the authors’ institute and literature articles were pooled and described. Survival analysis and multivariable Cox regression analysis were performed to evaluate therapies and investigate prognostic factors for PFS.

**Results:**

Our cohort included 22 females and 16 males, with a median age of 33 years. PSAE developed mostly on cervical and cervicothoracic levels. The median length measured 3 segments. Half of PSAE were intramedullary. Pain was the most common symptom. The median duration of symptoms was 6 months. Neurological statuses were improved in 76% following treatments, whereas clinical tumor progression occurred in 41.7%. The estimated median progression-free survival was 132 months, and the estimated median survival was 192 months. The median Ki-67 index was 15%. Patients aged less than or equal to 25 experienced worse neurological statuses and more repeated progression. Age less than or equal to 25 (HR 10.312, 95%CI 1.535-69.260, p=0.016), gross total resection (HR 0.116, 95%CI 0.020-0.688, p=0.018), and radiotherapy (HR 0.084, 95%CI 0.009-0.804, p=0.032) are three prognostic factors for tumor progression.

**Conclusion:**

Tumor progression remains a big concern in the clinical course of PSAE. Being aged above 25, undergoing GTR, and accepting adjuvant radiotherapy put patients at lower risk for tumor progression. Younger patients might have worse neurological statuses compared with those aged over 25.

## Introduction

The updated annual age-adjusted incidence rate of primary intraspinal tumors was 0.74 per 100,000 (1). Of these, intraspinal ependymomas are the predominant type in children and adolescents, and the most common intramedullary tumors in adults (1–3). According to the 2016 World Health Organization (WHO) classification, primary spinal anaplastic ependymomas (PSAE) correspond to grade III tumors, accounting for 2.2%-7.3% of intraspinal ependymomas (2–5). Distinct from dissemination of intracranial anaplastic ependymomas, PSAE arise initially within the spinal cord, or in limited cases, intradural extramedullary (IDEM) space (6, 7).

Since MØRK S. J. reported the first patient, 57 PSAE cases have been recorded in literature (8). The clinical outcomes varied considerably from long-term survival without progression to death resulting from repeated recurrence or metastases (9, 10). Gross total resection (GTR) may be beneficial to patients (5). Surgery was therefore performed as the first-line treatment in all known cases (10). Moreover, adjuvant radiotherapy and chemotherapy, including temozolomide, were also trialed (11). Nevertheless, the effectiveness of these treatments remains unclear. Our study therefore aims to investigate the efficacy of various therapies and explore the possible prognostic factors for tumor progression.

## Materials and methods

### Population and study strategy

From December 2008 to August 2020, a total of 38 patients pathologically diagnosed with PSAE were included consecutively at one single institute. The inclusion criteria were (1) primary intraspinal tumor, and (2) the definitive histopathology demonstrating increased mitotic count, high cell density, extensive microvascular proliferation and necrosis in an ependymal tumor based on WHO central nervous system tumor classification(2). The exclusion criteria were (1) identified dissemination from intracranial anaplastic ependymoma, (2) existence of previous surgery from other institutes, and (3) refusal or inability to give informed consent. Magnetic resonance imaging (MRI) of the whole neuraxis was performed to exclude possible drop metastases. We studied parameters including age at diagnosis, gender, symptoms, duration of symptoms, location and length of the lesion, pre- and postoperative neurological statuses, treatments, Ki-67 index, surgical morbidity, frequency of tumor progression, mortality causes, progression-free survival (PFS) and overall survival (OS) (**Supplement Material 1**). PFS was defined as the survival time without evidence of recurrence or metastasis since the diagnosis, and OS was the duration from diagnosis to death caused by PSAE. Frequency of tumor progression was defined as the number of times that PSAE progressed. Follow-ups were performed every six months within the first two years after discharge and then done yearly through out-patient. The last follow-up date was June 19th, 2021. Censoring occurs when no endpoint event happens till the last follow-up, or participants drop out of the cohort due to reasons irrelevant to the disease. GTR was defined as resection with no visual residue and no postoperative contrast-enhancing tumor. Subtotal resection was defined as resection with residue volume less than 10% on MRI, and partial resection was defined for the rest. Modified McCormick classification (MMC) was applied to assess neurological statuses (**Supplement Material 2**) (12). Strengthening the Reporting of Observational studies in Epidemiology protocol was followed across the retrospective cohort study. This study was approved by the Institutional Review Board of our hospital, and all the patients and their kins have consented to the submission and publication of the study.

### Literature search strategy and study eligibility

**Figure 1:**
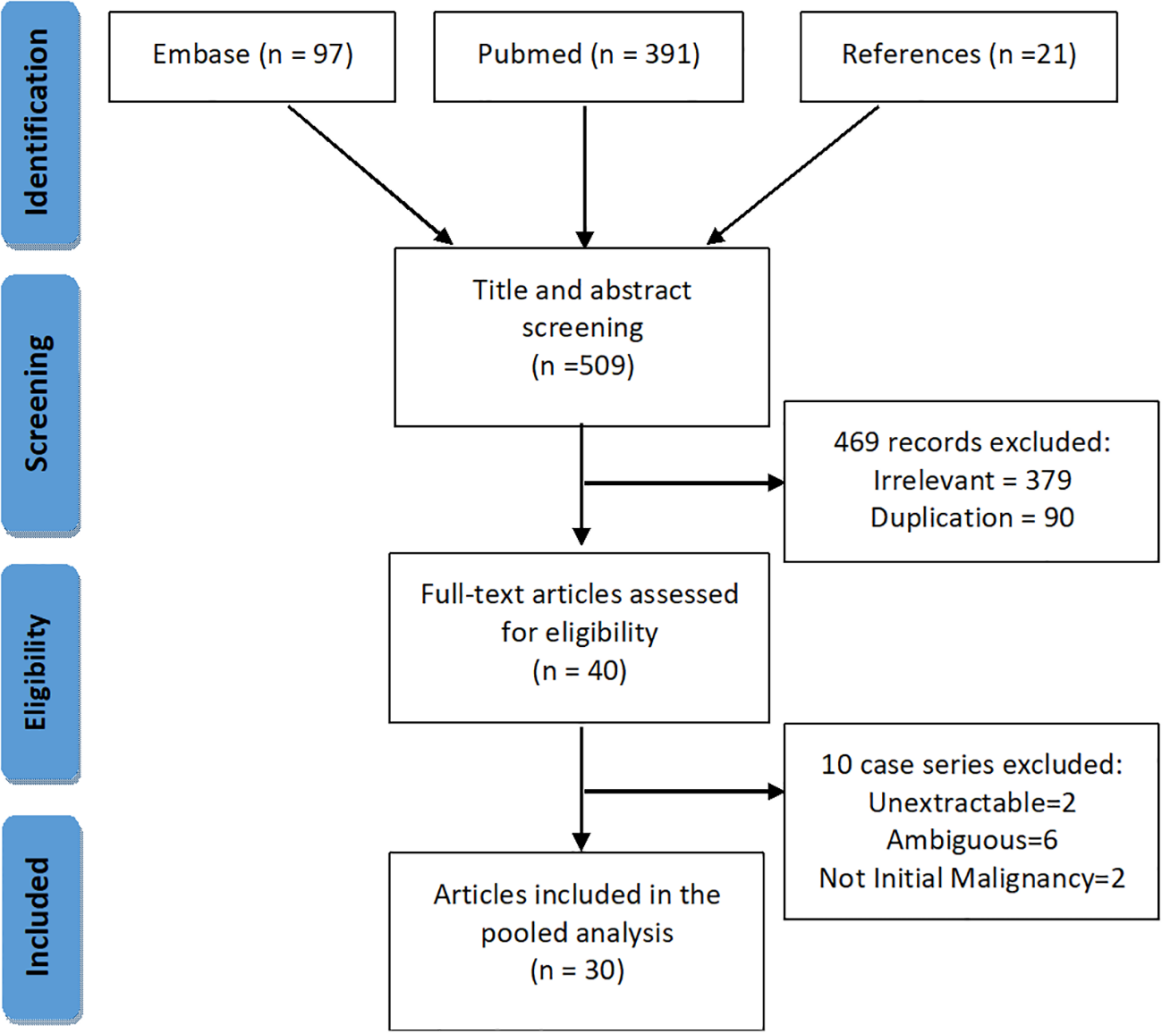
PRISMA chart showing the inclusion and the exclusion process for the literature articles.

We performed a systematic search of PSAE on PubMed and EMBASE (**Figure 1**). Keywords and MeSH terms, such as “spinal cord tumor”, “ependymal tumor”, “primary spinal anaplastic ependymoma” were incorporated into our search strategy. We also searched the references of the included articles for possible cases.

Two reviewers independently and in duplicate performed the title and abstract screening, full-text eligibility assessment and data extraction for every retrieved item from inception till January 1^st^, 2021 (**Supplement Material 3**). A third reviewer was consulted if there was any ambiguity. Inclusion criteria for literature cases were (1) primary intraspinal tumor, and (2) the definitive diagnosis of spinal anaplastic ependymoma. Exclusion criteria were (1) undefined pathological diagnosis or ambiguous definitions, such as “grade 4 ependymoma”, “high-grade ependymoma”, and “poorly differentiated”, and (2) intraspinal dissemination secondary to intracranial anaplastic ependymomas. Preferred Reporting Items for Systematic Reviews and Meta-Analyses protocol was adhered to throughout the search (13).

### Risk of bias assessment

Risk of bias was assessed through the Joanna-Briggs Institute Critical Appraisal tools for case reports and case series (**Supplement Materials 4, 5**) (14, 15). We noticed reporting bias when including studies. Therefore, we compared authors, institutes, and cases to avoid duplication. Besides, there was attrition bias of critical data, such as treatment and prognosis, and we sent request emails to authors for the missing data.

### Statistical analysis

The clinical characteristics, therapies, and outcomes of PSAE cases from two age groups were compared using various non-parametric tests. Survival analyses were executed using Kaplan-Meier method, and group comparisons were tested with log-rank test. We conducted Schoenfeld residual analysis to meet the proportional hazard assumption and then employed multivariable Cox regression analysis to explore the possible prognostic factors. Hazard radio (HR) and 95% confidence interval (CI) were presented. *P*-value< 0.05 was defined statistically significant. The cut-points of age and Ki-67 index were selected using X-Tile (version 3.6.1, Yale University 2003-05) and R package (survminer version 0.4.9, provided by Alboukadel Kassambara). Statistical analyses and graph-plotting were performed using R (version 4.1.1; R Development Core Team) and GraphPad Prism (GraphPad Software, San Diego, California). A statistician was consulted for all the medical statistics we used.

## Results

### Clinical characteristics

Thirty-eight PSAE cases identified by our pathologists were included from December 2008 to August 2020 (**Table 1**). Initial symptoms were missing in five patients, the surgical charts were unavailable in four, and the adjuvant therapy records were missing in four. Two patients were lost to follow-up after discharge.

**Table 1:** The clinical features, interventions, and outcomes of PSAE in our hospital and from literature.

Characteristics	Our hospital (N=38)	Literature (N=57)
Demographics		
Median age (years)	33 (7-61)	30 (2-67)
Male (%)	16 (42.1%)	33 (61.1%)
Location (%)	n=38	n=54
Cervical	10 (26.3%)	13 (24.1%)
Cervicothoracic	10 (26.3%)	6 (11.1%)
Thoracic	7 (18.4%)	14 (25.9%)
Lumbar	9 (23.7%)	15 (27.8%)
Multiple	2 (5.3%)	6 (11.1%)
Site of Tumors (%)	N=38	n=52
Intramedullary	19 (50%)	24 (46.2%)
Exophytic	6 (15.8%)	4 (7.7%)
IDEM	13 (34.2%)	24 (46.2%)
Median number of segments	3 (1-9)	4 (1-9)
Symptoms (%)	n=33	n=43
Pain	27 (81.8%)	29 (67.4%)
Sensory Deficit	24 (72.7%)	30 (69.8%)
Limb Weakness	20 (60.6%)	33 (76.7%)
Sphincter Dysfunction	12 (36.4%)	21 (48.8%)
Median duration of symptoms (months)	6 (0.33-24) (n=29)	4 (0.25-48) (n=37)
Gross Total Resection (%)	25 (73.5%) (n=34)	32 (60.4%) (n=53)
Radiotherapy (%)	17 (50%) (n=34)	34 (75.6%) (n=45)
Chemotherapy (%)	5 (14.7%) (n=34)	8 (17.8%) (n=45)
Ki-67 index	15% (3%-90%)	NA
Median follow-up time (months)	65 (10-276) (n=36)	31 (0.2-236.4) (n=47)
Frequency of Progression	n=36	n=47
0 (%)	21 (58.3%)	21 (44.7%)
1	8	18
≥2	7	8
EMST (months) (95%CI)	192 (63-321) (n=36)	95 (47-143) (n=47)
5-year survival rate	96%	60%
10-year survival rate	79%	43%
EMPFT (months) (95%CI)	132 (accurate) (n=36)	36 (13-59) (n=47)
1-year PFS rate	94.3%	82%
3-year PFS rate	72.4%	51%
5-year PFS rate	57.4%	39%

CI, confidence interval; EMPFT, estimated median progression-free time; EMST, estimated median survival time; IDEM, intradural extramedullary; NA, not available; PFS, progression-free survival.

**Figure 2:**
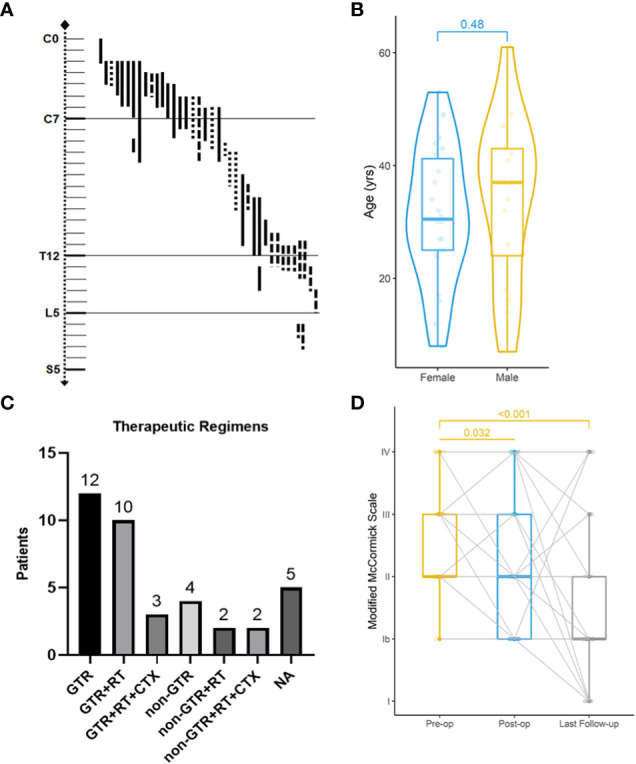
Clinical features, treatments, and neurological improvement of 38 patients from our institute. Localization of primary spinal anaplastic ependymomas **(A)**. **──***, intramedullary;* **……** *exophytic;* **−·−·** *intradural extramedullary*. Age distribution of gender-based subgroups (Wilcoxon rank sum test) **(B)**. Various combinations of therapies **(C)**. *GTR gross total resection; RT radiotherapy; CTX chemotherapy; NA not available.* Preoperative (pre-op), postoperative (post-op) and the latest Modified McCormick classification of 38 patients (Friedman test) **(D)**.

**Figure 3:**
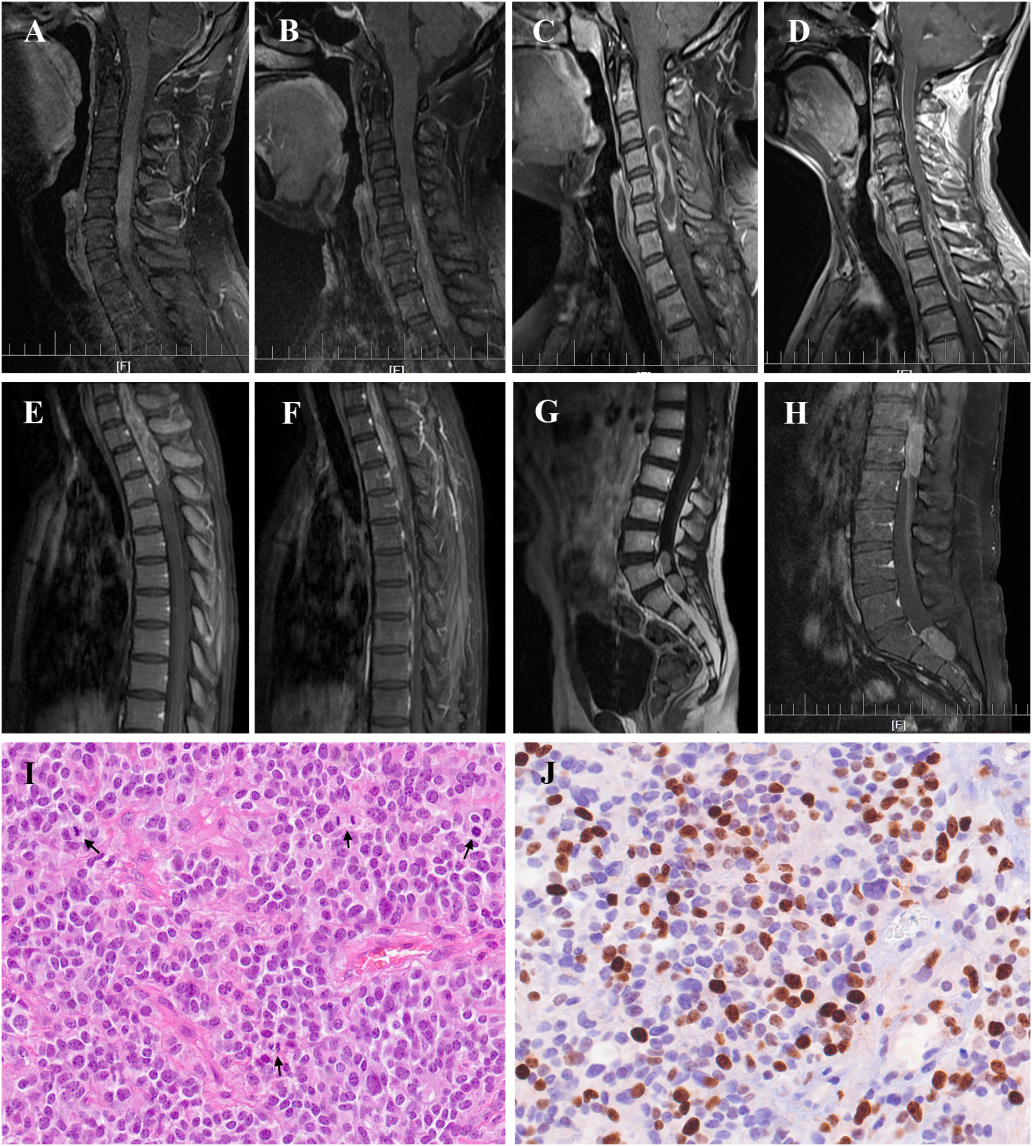
Radiological manifestation and histopathology of primary spinal anaplastic ependymomas (PSAE). Magnetic Resonance Imaging of PSAE revealed heterogenous or homogenous contrast-enhancing tumors at various locations within the spinal canal **(A–H)**. Histopathology of PSAE depicted increased cellularity, brisk mitotic activity, and poor cellular differentiation among typical ependymal tumor features, **(I)** Hematoxylin and eosin (H&E) 400×; **(J)** Ki-67 staining 400×. *Black arrows* indicated the mitotic phases in **(I)**.

With a median age of 33 (range 7-61 years), 16 males and 22 females were identified. Age distribution was comparable between males and females (**Figure 2**). The most common symptom was pain, followed by sensory deficits, limb weakness and sphincter dysfunction. The duration of symptoms varied from 0.33 month to 24 months. Preoperatively, approximately 52% (total=33) patients were classified as MMC grade II, followed by 33% as grade III, 12% as grade IV, and 3% as grade Ib (**Supplement Material 1**). Twenty cases of PSAE occurred to cervical and cervicothoracic areas. Thoracic cord was affected in seven cases, the conus medullaris and below area was involved in nine cases, and multiple lesions occurred to two patients. The median length of tumors measured three segments (range 1-9). Verified by surgical charts, 50% (total=38) PSAE were intramedullary, 15.8% had exophytic portion, and 34.2% occurred in IDEM space. On MRI, PSAE manifested itself as either heterogeneous or homogenous contrast-enhancing mass. Tumoral cysts could also be observed rostrally or caudally (**Figure 3**). The pathological reports revealed the median percentage of Ki-67 index was 15% (range 3%-90%).

Fifty-seven cases of PSAE were identified in literature, and the clinical data were pooled (**Supplement Material 2**). Incomplete descriptions of PSAE were common, and Ki-67 index was unavailable in literature.

### Therapeutic regimens

Four surgical charts were missing as mentioned, including one of the patients who dropped out of the cohort. Thirty-four patients underwent surgery initially, of which 25 achieved GTR (**Figure 2**). Six patients had subtotal resection, two achieved partial resection, and one who was lost to follow-up had excisional biopsy. Seventeen patients accepted the adjuvant radiotherapy, while the other seventeen declined it. Chemotherapy was administered in five cases with three given temozolomide. With respect to the treatment regimens, GTR alone was performed in twelve, GTR followed by radiotherapy in ten, and the combination of GTR, radiotherapy and chemotherapy in three. For patients with non-GTR, two underwent adjuvant radiotherapy and two adopted both radiotherapy and chemotherapy. The treatment regimen could not be summarized in four patients without surgical charts and one case that was lost to follow-up.

In literature, GTR was performed in 60.4% cases (total=53), adjuvant radiotherapy was applied in 75.6% (total=45), and various chemotherapies were administered in 17.8% (total=45) cases (**Table 1**).

### Clinical outcomes

The median follow-up of our cohort was 65 months (range 10-276 months). Postoperatively, two patients experienced central nervous system infection, two had poor incision healing, and one developed hallucination. Others experienced no surgical complication. At discharge, MMC grades were improved in 67% (total=33) patients, unchanged in 21%, and worsened in 12%. During the last follow-up, MMC grades were improved in 76%, unchanged in 12%, and worsened in 12%. The neurological improvement was significant postoperatively (p=0.032) and at last follow-up (p<0.001).

Clinical progression occurred in 41.7% (total=36) patients, including 11 with recurrence and four with both recurrence and metastases. Eight patients had one recurrence, while seven experienced repeated recurrences after reoperations. Four patients died of PSAE metastases. The estimated median progression-free time (EMPFT) was 132 months, and the estimated median survival time (EMST) was 192 (95%CI 63-321) months. About 94.3% of patients survived one year without tumor progression, 72.4% survived three years, and 57.4% survived five years. Overall, 96% of patients survived five years, and 79% survived ten years.

The median follow-up time of literature cases was 31 (0.2-236.4) months. Approximately, 55.3% of cases experienced clinical progression, of which 17% cases were affected more than once. The EMST of literature cases was 95 (47-143) months.

### Prognostic factors

**Figure 4:**
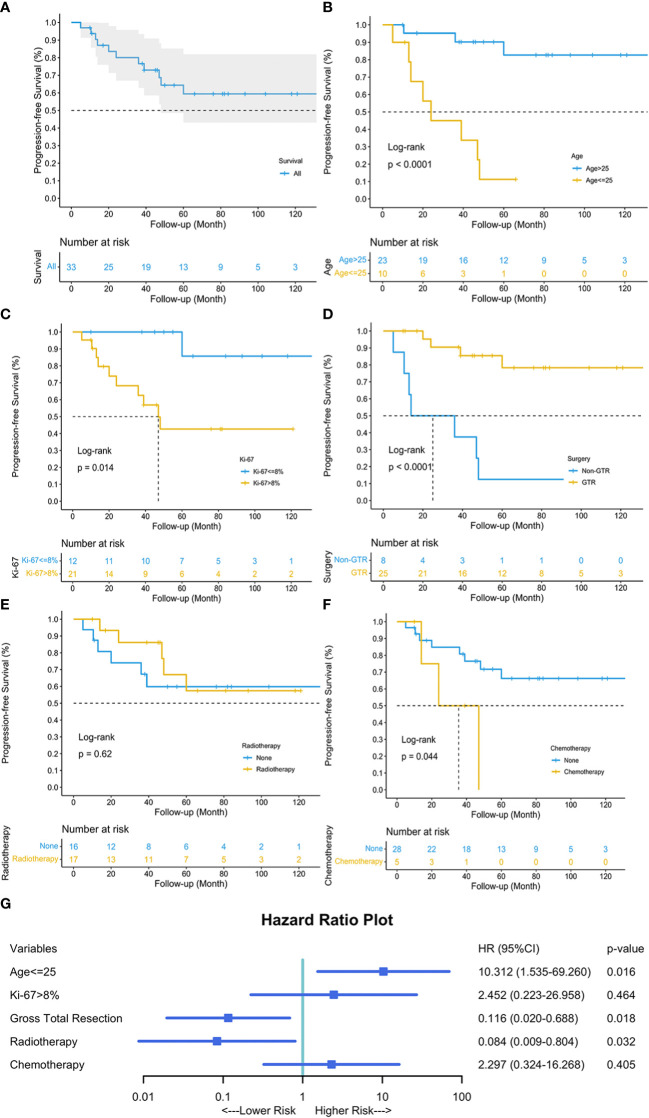
Kaplan-Meier curve depicted the progression-free survival of our 38 patients **(A)**. Univariable survival analyses of age **(B)**, Ki-67 **(C)**, surgery **(D)**, radiotherapy **(E)**, and chemotherapy **(F)** were visualized, respectively. Multivariable Cox Regression analysis revealed that age less than or equal to 25, GTR and adjuvant radiotherapy were three independent prognostic factors for tumor progression **(G)**. *Blue square* indicated the hazard ratio (HR) values, and *error bars* revealed the 95% confidence intervals.

Univariable survival analysis revealed that age above 25 (p<0.0001), Ki-67 ≤ 8% (p=0.014), GTR (p<0.0001), and chemotherapy (p=0.044) were significant variables, while radiotherapy (p=0.62) showed otherwise (**Figure 4**). The multivariable Cox regression analysis revealed that GTR (HR 0.116, 95%CI 0.020-0.688, *p*=0.018) and adjuvant radiotherapy (HR 0.084, 95%CI 0.009-0.804, *p*=0.032) reduced the risk of tumor progression, while age less than or equal to 25 years old (HR 10.312, 95%CI 1.535-69.260, *p*=0.016) increased the risk.

The cutpoint of the continuous variable, age, was calculated through X-Tile and verified by R package. Patients aged above 25 tended to have different tumor locations (p=0.019), less limb weakness (p=0.049), and better preoperative neurological status (p=0.019) (**Table 2**). Considering the clinical outcomes, younger group (age ≤ 25) seemed to have worse neurological statuses (p=0.008), more tumor progression (p=0.002), more frequent progression (p=0.005), and worse progression-free survival (p<0.0001).

**Table 2:** Characteristics of primary spinal anaplastic ependymoma in patients above and below 25.

Characteristics	Age≤25 (N=11)	Age>25 (N=27)	*p*-value
Female (%)	7 (63.6%)	15 (55.6%)	0.729^a^
Location (%)			**0.019** ^a^
Cervical	0	10 (37.0%)	
Cervicothoracic	4 (36.4%)	6 (22.2%)	
Thoracic	5 (45.5%)	2 (7.4%)	
Lumbar	2 (18.2%)	7 (25.9%)	
Multiple	0	2 (7.4%)	
Median number of segments	4 (2, 6)	3 (1, 9)	0.166^b^
Site of Tumors (%)			0.494^a^
Intramedullary	5 (45.5%)	14 (51.9%)	
Exophytic	3 (27.3%)	3 (11.1%)	
IDEM	3 (27.3%)	10 (37.0%)	
Median duration of Symptoms (months)	6 (1, 24)	7 (0.33, 24)	0.536^b^
Symptoms (%)	n=10	n=23	
Pain	8 (80.0%)	19 (82.6%)	1^a^
Limb Weakness	9 (90.0%)	11 (47.8%)	**0.049^a^**
Sensory Deficit	7 (70.0%)	17 (73.9%)	1^a^
Sphincter Dysfunction	4 (40.0%)	8 (34.8%)	1^a^
Pre-op MMC (%)	n=10	n=23	**0.019^a^**
Ib	0	1 (4.3%)	
II	2 (20.0%)	15 (65.2%)	
III	7 (70.0%)	4 (17.4%)	
IV	1 (10.0%)	3 (13.0%)	
Gross Total Resection (%)	5 (50.0%) (n=10)	20 (83.3%) (n=24)	0.085^a^
Radiotherapy (%)	5 (45.5%)	12 (52.2%) (n=23)	1^a^
Chemotherapy (%)	3 (27.3%)	2 (8.7%) (n=23)	0.300^a^
Ki-67 index	20% (8%-30%)	10% (3%-90%)	0.135^b^
Post-op MMC			**0.008^a^**
Last follow-up MMC			**0.008^a^**
Frequency of Progression, median	1 (0, 4)	0 (0, 3) (n=25)	**0.005^b^**
Tumor Progression (%)	9 (81.8%)	6 (24.0%) (n=25)	**0.002^a^**
EMPFT (95%CI, months)	24 (14, NA)	132 (not reached)	**<0.0001^c^**
1-year PFS rate	90.9%	95.7%	
3-year PFS rate	40.4%	86.5%	
5-year PFS rate	10.1%	73.7%	
Mortality	2 (18.2%)	2 (8.0%) (n=25)	0.571^a^
EMST (95%CI, months)	101 (101, NA)	192 (192, NA)	0.130^c^

CI, confidence interval; EMPFT, estimated median progression-free time; EMST, estimated median survival time; IDEM, intradural extramedullary; MMC, Modified McCormick; NA, not available; PFS, progression-free survival; PSAE, primary spinal anaplastic ependymoma.

a. Fisher’s exact test; b. Wilcox rank sum test; c. Log-rank test.

## Discussion

The newly published 5^th^ edition of the WHO classification of central nervous system tumors introduced a new classification of ependymal tumors based on molecular diagnostics (16). Ependymomas located inside the spinal canal were classified into two subtypes- spinal ependymoma, and spinal ependymoma, *MYCN*-amplified (SP-EPN-MYCN) (16). However, the major changes remain consistent with the established histopathological diagnostics, and anaplastic ependymomas still play a critical role in the integrated diagnosis.

PSAE are a rare intraspinal tumor, and little is known about its clinical features, therapies, and prognosis (5, 10). We reported the largest retrospective cohort of 38 patients to date, and systematically reviewed 57 cases from literature. Additionally, we explored the efficacy of various interventions and identified the prognostic factors for tumor progression.

### Clinical characteristics

Liu et al. observed distinctive male predominance in their sample, which was consistent with our review (5). However, our single-institute sample revealed otherwise. PSAE seemed to affect mainly a young population (median age, 33 years), similar to the sample pooled from literature (median age, 30 years). Some investigations favored the finding and showed grade III ependymomas, including the anaplastic subtype, occurred to children more than adults (17). A more intriguing fact was that young age seemed a critical factor that negatively affected the prognosis. We noticed younger population (age ≤ 25) had worse neurological statuses before and after surgery, and they tended to have a larger tumor progression rate and higher frequency of progression. We also observed the progression-free survival was severely compromised. Courtney et al. ran a multivariable analysis on the clinical features of ependymomas among children and adolescents and concluded that a younger age indicated worse 5-year survival (18). Though they discussed no survival concerning tumor progression, this might indicate that younger age portends a general worse prognosis.

Resembling all subtypes of intraspinal ependymomas, local pain was the most common symptom, followed by sensory deficit, limb weakness and sphincter dysfunction (3, 12). However, among intraspinal ependymomas of all WHO grades, the symptom duration of PSAE patients was shorter, which could possibly be attributed to its malignant behavior (median duration of symptoms, 6 months versus 19-32 months) (3, 12). Furthermore, with respect to the preoperative neurological statues, 45% of our patients were graded MMC III/IV, whereas, according to one large multicenter cohort, only 7% of intraspinal ependymomas were graded within this range (3). We believe the number again reflected the aggressiveness of PSAE.

PSAE affected mostly the cervical and cervicothoracic cord, measuring a median length of 3 segments, which resembled the results of a previous cohort (5). However, it seemed in younger patients (age ≤ 25), PSAE tended to occur in lower levels other than cervical canal. On MRI, PSAE showed no identifying features from other intraspinal ependymomas (19). We therefore tried to find some useful features. A multicenter cohort revealed that 36% (total=158) intraspinal ependymomas occurred in intradural extramedullary space (3). In our sample, half of PSAE developed exophytic portion or in IDEM space, consistent with the constituent of literature cases. A literature review found 30% (total=40) intradural extramedullary ependymomas were anaplastic. However, all studies failed to conclude the association between PSAE and ependymomas in IDEM space (3).

The new malignant subtype, SP-EPN-MYCN, were mainly (11/12) classified into WHO grade III ependymoma (20). Intriguingly, it mostly affected adolescents and young adults, presented with exophytic growth pattern or IDEM localization, and showed aggressive behavior (20, 21).We reasonably suspected that some of our cases might have MYCN amplification. However, we hardly conducted the methylome analysis due to the retrospective study design. Instead, we performed Ki-67 staining for every PSAE. Ki-67 was an independent prognostic factor for both OS and PFS, and a 7% cut-off could classify ependymomas of all locations into two-tiered grades (22). We used X-Tile and a R package to find the best cut-off value in our sample, which was 8% of Ki-67 index. Though our survival analysis found Ki-67 over 8% indicated worse PFS, the multivariable Cox regression revealed that Ki-67 was not a significant prognostic factor.

### Therapeutic strategies

The treatment algorithms changed over the past few years (23). However, surgery is still the first-line recommendation in the treatment strategy of intraspinal ependymomas, and a radical resection has been shown to favor progression-free survival (5, 24). We also found an improved PFS following GTR, and the GTR surprisingly reduced the tumor progression by 88.4% (95%CI 0.020-0.688, p=0.018). The percentage of GTR for intraspinal ependymomas ranged distinctively from 11% to 89% in the published studies (3, 25, 26). The GTR rate was 73.5% in our sample and 61.5% in our systematic review. As opposed to infiltrative spinal gliomas, intraspinal ependymomas are usually well-demarcated during microsurgery. Liu et al. also found that, similar to lower grade ependymomas, the interface between PSAE and the spinal cord tended to be well-defined instead of diffusely infiltrative, which was consistent with our findings (5). Additionally, ependymomas in IDEM space were frequently encapsulated with a mere microvascular plane connected with central nervous tissues, which again favors a radical resection (27, 28). However, we hardly found the correlation between tumor location and GTR in our cohort (Pearson contingency coefficient 0.263, p=0.112). Nevertheless, we think GTR holds the key to a better outcome.

The role of radiotherapy remains controversial (5). Generally, radiotherapy has been recommended as a surgical adjunct where GTR is not possible (29). Some investigators also revealed that adjuvant radiotherapy could reduce recurrence among low-grade spinal ependymomas (25, 30). Given the malignancy of PSAE, we routinely recommended the adjuvant radiotherapy: the radiation fields included the tumor with 2cm margin above and below the tumor bed, and the total dose ranged from 45-60 Gy. However, only half the patients accepted the treatment, and the rest half declined it due to various reasons, such as economic problems or unwillingness to take the radiation risks. Through multivariable analysis, we found local radiotherapy considerably reduced tumor progression by 91.6% (95%CI 0.009-0.804, p=0.032). In our cohort, four patients died of intracranial metastases. Similarly, the leptomeningeal dissemination (27), intracranial metastases (31), and even bone metastases (31), were also frequently reported in literature. Lin et al. found the salvage radiotherapy could improve life quality for patients with intraspinal ependymomas suffering from local recurrence or leptomeningeal seeding (32). Local radiotherapy was common, while some also proposed for craniospinal radiation (33). Conversely, one study argued craniospinal radiotherapy was no beneficial to intracranial anaplastic ependymoma patients (34). Therefore, we believe local radiotherapy could be given before craniospinal radiation was proved effective and safe. Based on literature reviews and our personal experience, we propose the radiation dose could be trialed between 45-60Gy (25, 35).

Chemotherapy was rarely administered across any PSAE reports. It was not a standard recommendation, and only sparse case reports trialed it (11, 36). Unfortunately, we failed to conclude anything favoring the use of chemotherapy. In our cohort, five patients were administered with chemotherapy, and three were given temozolomide. Of the three, one experienced local recurrence at 47 months. Temozolomide is an oral alkylating agent, which was reported to modulate the tumor resistance through depleting O6-methylguanine-DNA methyltransferase (MGMT) activity (37). Lack of MGMT promotor hypermethylation and high expression of MGMT were observed in 75% (total=12) of recurrent anaplastic ependymomas, which theoretically favored the trial of temozolomide in PSAE patients (38). A recent phase II study of temozolomide declared the effectiveness against the recurrence in low-grade and anaplastic ependymomas (39). This study included 7 PSAE cases and might be conducive to future treatment choices.

### Clinical outcomes and prognostic factors

The overall prognosis of PSAE was unfavorable. In literature, 60% of patients survived the 5^th^ year and 43% survived the 10^th^. After various combinations of therapies, the MMC of our patients improved considerably. We owe this to the radical resection, standardized postoperative care, and close follow-up. The pooled literature cases instead underwent heterogenous surgical interventions, radiotherapies, and supportive therapies, which might have led to a worse general outcome. What concerned us most was the clinical progression. It occurred in 41.7% of patients in our cohort and 55.3% in literature. A single-institute study reported the recurrence rate of intraspinal ependymomas was 50%, and two recurrent cases were diagnosed as late as 14 and 20 years (9). A long-term follow-up therefore seemed necessary. Another study argued the PFS for anaplastic ependymomas was shorter than that of the benign ones (40). The high progression rate thus necessitated the investigation into the correlated prognostic factors.

Three prognostic factors for PFS were identified through our cohort. As mentioned above, GTR and radiotherapy were deemed protective factors with respect to tumor progression. Some investigators also found the critical role of GTR in preventing PSAE progression, though they failed other investigation due to limited sample size (5). In our cohort, we identified the cutpoint of 25 years old through X-Tile and R package using our sample and found that an age less than or equal to 25 put patients at increased risk of tumor progression (HR 10.312, 95%CI 1.535-69.260, p=0.016). A study on the Surveillance Epidemiology End Results database (1973-2003) found increasing age was significantly associated with better survival. This age-related prognostic effect might similarly affect PSAE patients.

### Limitations

Three limitations of this study need to be stated. Firstly, the sample size was still small, leading to a wide confidence interval. Secondly, no methylome analysis was conducted, and no SP-EPN-MYCN subtype was identified in our retrospective cohort. Thirdly, we systematically extracted available cases, but the risk of reporting bias might potentially impact the results. However, PSAE is a rare entity, and this study answered some clinical questions.

## Conclusion

Tumor progression remains a big concern in the clinical course of PSAE. Being aged above 25, undergoing GTR, and accepting adjuvant radiotherapy put patients at lower risk for tumor progression. Younger patients might have worse neurological statuses compared with those aged over 25.

## Data availability statement

The original contributions presented in the study are included in the article/**Supplementary Material**. Further inquiries can be directed to the corresponding author.

## Author contributions

Conceptualization, LWu, LWa, WZ, YX; Data curation, WJ, JY, LWa, WZ; Methodology, LWa, LWu; Visualization, LWu, LWa; Writing-original draft, LWa, LiWu; Writing-review & editing, YX, LWu, LWa, WZ.
