# Supplementary material for: Primary spinal anaplastic ependymoma: A single-institute retrospective cohort and systematic review
Source: Front Oncol. 2023 Feb 7;13:1083085. doi: 10.3389/fonc.2023.1083085 (PMC9941548; doi:10.3389/fonc.2023.1083085)
Supplement: Supplementary file 1 [file Table_1.docx]

**Supplement Material 1. The Data of 38 Patients Diagnosed with Primary Spinal Anaplastic Ependymoma at Our Center.**

| Case No. | Age (yr) | Gender | Pain | Weakness | Sensory | Sphincter | Pre-op MMC | Duration of symptoms (mos) | Location | Segments | Ki-67 | Site |
| --- | --- | --- | --- | --- | --- | --- | --- | --- | --- | --- | --- | --- |
| 29 | 7 | male | 1 | 1 | 0 | 0 | 3 | 1 | Thoracic | 2 | 0.20 | IDEM |
| 32 | 8 | female | NA | NA | NA | NA | NA | NA | Cervicothoracic | 4 | 0.20 | Intramedullary |
| 5 | 12 | female | 1 | 1 | 1 | 1 | 4 | 1.5 | Thoracic | 4 | 0.30 | Intramedullary |
| 27 | 14 | male | 1 | 1 | 1 | 1 | 3 | 3 | Thoracic | 3 | 0.10 | Exophytic |
| 9 | 16 | male | 1 | 1 | 1 | 0 | 3 | 6 | Thoracic | 6 | 0.20 | Intramedullary |
| 38 | 16 | female | 0 | 1 | 0 | 0 | 3 | 6 | Cervicothoracic | 3 | 0.20 | Intramedullary |
| 13 | 17 | female | 1 | 1 | 1 | 0 | 3 | 12 | Lumbar | 4 | 0.10 | IDEM |
| 11 | 18 | male | 1 | 1 | 0 | 0 | 2 | 24 | Lumbar | 5 | 0.08 | IDEM |
| 15 | 24 | female | 0 | 1 | 1 | 1 | 3 | 5 | Cervicothoracic | 4 | 0.30 | Exophytic |
| 4 | 25 | female | 1 | 1 | 1 | 0 | 2 | 6 | Thoracic | 5 | 0.30 | Exophytic |
| 34 | 25 | female | 1 | 0 | 1 | 1 | 3 | NA | Cervicothoracic | 3 | 0.15 | Intramedullary |
| 14 | 26 | male | 1 | 0 | 1 | 0 | 2 | 12 | Thoracic+Lumbar | 6 | 0.08 | IDEM |
| 7 | 27 | female | 0 | 1 | 0 | 0 | 2 | NA | Cervicothoracic | 9 | 0.20 | Intramedullary |
| 22 | 27 | female | 1 | 1 | 1 | 0 | 3 | 6 | Cervical | 3 | 0.08 | Intramedullary |
| 1 | 30 | female | NA | NA | NA | NA | NA | NA | Lumbar | 2 | 0.10 | IDEM |
| 37 | 30 | female | 1 | 1 | 1 | 1 | 4 | 2 | Cervical+Thoracic | 6 | 0.90 | Intramedullary |
| 17 | 31 | female | 1 | 0 | 1 | 0 | 2 | 3 | Cervical | 4 | 0.30 | Intramedullary |
| 2 | 32 | female | NA | NA | NA | NA | NA | NA | Lumbar | 2 | 0.30 | IDEM |
| 3 | 32 | male | NA | NA | NA | NA | NA | NA | Lumbar | 2 | 0.40 | IDEM |
| 19 | 34 | male | 1 | 0 | 1 | 0 | 2 | 2 | Cervical | 4 | 0.15 | Intramedullary |
| 20 | 34 | female | 1 | 1 | 0 | 0 | 2 | 2 | Thoracic | 1 | 0.08 | Exophytic |
| 8 | 37 | male | 0 | 1 | 0 | 0 | 2 | NA | Lumbar | 4 | 0.15 | IDEM |
| 23 | 37 | female | 1 | 0 | 1 | 0 | 2 | 0.33 | Cervicothoracic | 3 | 0.15 | Intramedullary |
| 24 | 37 | male | 1 | 0 | 0 | 0 | 2 | 6 | Cervicothoracic | 6 | 0.08 | IDEM |
| 28 | 39 | female | 1 | 0 | 1 | 0 | 2 | 12 | Lumbar | 2 | 0.20 | IDEM |
| 21 | 41 | male | 1 | 0 | 1 | 0 | 2 | NA | Cervical | 6 | 0.10 | Intramedullary |
| 10 | 42 | male | 1 | 1 | 1 | 0 | 2 | 3 | Cervical | 2 | 0.10 | Intramedullary |
| 16 | 42 | female | 1 | 0 | 0 | 0 | 2 | 2 | Cervical | 2 | 0.04 | Intramedullary |
| 6 | 43 | female | 0 | 0 | 1 | 1 | 2 | 8 | Cervical | 3 | 0.05 | Intramedullary |
| 35 | 43 | male | 1 | 1 | 1 | 1 | 2 | 10 | Thoracic | 2 | 0.08 | Intramedullary |
| 36 | 43 | male | 1 | 1 | 1 | 1 | 3 | 6 | Cervical | 2 | 0.06 | IDEM |
| 18 | 44 | female | NA | NA | NA | NA | NA | NA | Cervical | 2 | 0.50 | Exophytic |
| 30 | 45 | female | 1 | 0 | 1 | 0 | 2 | 12 | Cervicothoracic | 5 | 0.10 | Intramedullary |
| 12 | 47 | male | 1 | 0 | 0 | 0 | 1b | 9 | Cervical | 3 | 0.20 | Intramedullary |
| 25 | 49 | female | 1 | 0 | 1 | 1 | 3 | 12 | Cervicothoracic | 4 | 0.40 | Exophytic |
| 31 | 49 | male | 1 | 1 | 1 | 1 | 4 | 8 | Lumbar | 2 | 0.05 | IDEM |
| 33 | 53 | female | 1 | 1 | 1 | 1 | 3 | 12 | Lumbar | 3 | 0.03 | IDEM |
| 26 | 61 | male | 0 | 1 | 1 | 1 | 4 | 24 | Cervicothoracic | 3 | 0.05 | Intramedullary |

| Case No. | Surgery | Radiotherapy | chemotherapy | Treatment | Post-op MMC | Frequency of Tumor Progression | Last FU MMC | PFS1(mos) | Outcome1 | OS(mos) | Outcome2 |
| --- | --- | --- | --- | --- | --- | --- | --- | --- | --- | --- | --- |
| 1 | NA | NA | NA | NA | NA | 1 | NA | 54 | 1 | 55 | 0 |
| 2 | NA | NA | NA | NA | NA | 2 | 4 | 27 | 1 | 41 | 1 |
| 3 | NA | NA | NA | NA | NA | NA | NA | NA | NA | NA | NA |
| 4 | NA | 0 | 0 | NA | 1b | 1 | NA | 22 | 1 | 102 | 0 |
| 5 | 1 | 0 | 0 | GTR | 2 | 1 | 1 | 20 | 1 | 99 | 0 |
| 6 | 1 | 0 | 0 | GTR | 1b | 0 | 1b | 104 | 0 | 104 | 0 |
| 7 | 1 | 1 | 0 | GTR+RT | 1b | 0 | 1b | 121 | 0 | 121 | 0 |
| 8 | 0 | 0 | 0 | non-GTR | 1b | 3 | 4 | 36 | 1 | 192 | 1 |
| 9 | 0 | 0 | 0 | non-GTR | 2 | 1 | 1b | 13 | 1 | 14 | 0 |
| 10 | 1 | 0 | 0 | GTR | 1b | 0 | 1 | 76 | 0 | 76 | 0 |
| 11 | 1 | 1 | 0 | GTR+RT | NA | 0 | 1 | 66 | 0 | 66 | 0 |
| 12 | 1 | 1 | 0 | GTR+RT | 1b | 0 | 1b | 46 | 0 | 46 | 0 |
| 13 | 0 | 1 | 0 | non-GTR+RT | 3 | 1 | 1 | 48 | 1 | 64 | 0 |
| 14 | 1 | 1 | 0 | GTR+RT | 1b | 1 | 1b | 60 | 1 | 60 | 0 |
| 15 | 0 | 1 | 1 | non-GTR+RT+TMZ | 2 | 1 | 3 | 47 | 1 | 56 | 0 |
| 16 | 1 | 0 | 0 | GTR | 1b | 0 | 1b | 38 | 0 | 38 | 0 |
| 17 | 1 | 1 | 1 | GTR+RT+CTX | 1b | 0 | 1b | 39 | 0 | 39 | 0 |
| 18 | 0 | NA | NA | NA | NA | NA | NA | NA | NA | 1 | NA |
| 19 | 1 | 1 | 0 | GTR+RT | 2 | 0 | 1b | 17 | 0 | 17 | 0 |
| 20 | 1 | 0 | 0 | GTR | 1b | 2 | 1b | 132 | 1 | 276 | 0 |
| 21 | 1 | 1 | 0 | GTR+RT | 1b | 0 | 1b | 121 | 0 | 121 | 0 |
| 22 | 1 | 1 | 0 | GTR+RT | 1b | 0 | 1b | 118 | 0 | 118 | 0 |
| 23 | 1 | 0 | 0 | GTR | 1b | 0 | 1b | 76 | 0 | 76 | 0 |
| 24 | 1 | 0 | 0 | GTR | 3 | 0 | 1b | 55 | 0 | 55 | 0 |
| 25 | 0 | 0 | 0 | non-GTR | 2 | 2 | 2 | 10.5 | 1 | 45 | 0 |
| 26 | 0 | 1 | 0 | non-GTR+RT | 4 | 0 | 3 | 93 | 0 | 93 | 0 |
| 27 | 0 | 1 | 1 | non-GTR+RT+TMZ | 2 | 1 | 4 | 14 | 1 | 86 | 0 |
| 28 | 1 | 1 | 0 | GTR+RT | 1b | 0 | 1b | 81 | 0 | 81 | 0 |
| 29 | 1 | 1 | 1 | GTR+RT+CTX | 2 | 4 | 4 | 24 | 1 | 72 | 1 |
| 30 | 1 | 0 | 0 | GTR | 1b | 0 | 1 | 82 | 0 | 82 | 0 |
| 31 | 1 | 0 | 0 | GTR | 4 | 0 | 2 | 84 | 0 | 84 | 0 |
| 32 | 1 | 0 | 0 | GTR | NA | 2 | NA | 39 | 1 | 39 | 0 |
| 33 | 1 | 1 | 0 | GTR+RT | 3 | 0 | 1b | 45 | 0 | 45 | 0 |
| 34 | 0 | 0 | 0 | non-GTR | 4 | 3 | 4 | 5 | 1 | 101 | 1 |
| 35 | 1 | 0 | 0 | GTR | 1b | 0 | 1b | 50 | 0 | 50 | 0 |
| 36 | 1 | 1 | 0 | GTR+RT | 4 | 0 | 2 | 10 | 0 | 10 | 0 |
| 37 | 1 | 1 | 1 | GTR+RT+TMZ | 4 | 0 | 4 | 10 | 0 | 10 | 0 |
| 38 | 1 | 0 | 0 | GTR | 4 | 0 | 1 | 11 | 0 | 11 | 0 |

**ESM_1**

The Clinical Data of 38 Patients Diagnosed with Primary Spinal Anaplastic Ependymoma at Our Center.

For symptoms and treatments, 1 represented “present” and 0 for “absent”. Specially, for surgery, 1 meant gross total resection, while 0 meant otherwise. Outcome1 represented the statuses of tumor progression, while outcome2 represented the statuses of mortality; 1 meant “the occurrence of the event”, while 0 meant “censored”.

*CTX, chemotherapies; FU, follow-up; GTR, gross total resection; IDEM, intradural extramedullary; MMC, modified McCormick classification; NA, not available; OS, overall survival; PFS, progression-free survival; Post-op, Postoperative; Pre-op, preoperative; RT, radiotherapy; TMZ, temozolomide.*
