# Supplementary material for: Primary spinal anaplastic ependymoma: A single-institute retrospective cohort and systematic review
Source: Front Oncol. 2023 Feb 7;13:1083085. doi: 10.3389/fonc.2023.1083085 (PMC9941548; doi:10.3389/fonc.2023.1083085)
Supplement: Supplementary file 2 [file Table_2.docx]

**Supplement Material 2 The Modified McCormick Classification**

| Grade | Definition |
| --- | --- |
| Ⅰ | Neurologically normal  Gait normal  Normal professional activity |
| Ⅰb | Tired after walking several kilometers  Running is impossible, or moderate sensorimotor deficit does not significantly affect the involved limb  Moderate discomfort in professional activity |
| Ⅱ | Presence of sensorimotor deficit affecting function of involved limb  Mild to moderate gait difficulty  Severe pain or dysesthetic syndrome impairs quality of life  Independent function and ambulation maintained |
| Ⅲ | More severe neurological deficit  Requires cane and/or brace for ambulation or maintains significant bilateral upper-extremity impairment  May or may not function independently |
| Ⅳ | Severe neurological deficit  Requires wheelchair or cane and/or brace with bilateral upper-extremity impairment  Usually not independent |
