# Supplementary material for: Primary spinal anaplastic ependymoma: A single-institute retrospective cohort and systematic review
Source: Front Oncol. 2023 Feb 7;13:1083085. doi: 10.3389/fonc.2023.1083085 (PMC9941548; doi:10.3389/fonc.2023.1083085)
Supplement: Supplementary file 3 [file Table_3.docx]

**Supplement Material 3. The Data Extracted from The Included Literature Articles**

| **Case No.** | **Age(yr)** | **Gender** | **Pain** | **Weakness** | **Sensory** | **Sphincter** |
| --- | --- | --- | --- | --- | --- | --- |
| 1 | 12 | male | 1 | 0 | 1 | 0 |
| 2 | 34 | female | NA | NA | NA | NA |
| 3 | 46 | male | NA | NA | NA | NA |
| 4 | 6 | female | 1 | 0 | 0 | 0 |
| 5 | 13 | NA | NA | NA | NA | NA |
| 6 | 23 | NA | NA | NA | NA | NA |
| 7 | 48 | NA | NA | NA | NA | NA |
| 8 | 24 | female | 1 | 1 | 1 | 1 |
| 9 | 13 | female | NA | NA | NA | NA |
| 10 | 15 | male | NA | NA | NA | NA |
| 11 | 18 | male | NA | NA | NA | NA |
| 12 | 15 | female | 1 | 0 | 0 | 0 |
| 13 | 33 | male | 0 | 0 | 1 | 0 |
| 14 | 44 | male | 0 | 1 | 0 | 0 |
| 15 | 44 | female | NA | NA | NA | NA |
| 16 | 6 | male | 1 | 1 | 1 | 1 |
| 17 | 20 | male | 1 | 1 | 1 | 1 |
| 18 | 21 | female | NA | NA | NA | NA |
| 19 | 56 | male | 1 | 0 | 1 | 0 |
| 20 | 29 | female | 1 | 1 | 1 | 1 |
| 21 | 10 | female | NA | NA | NA | NA |
| 22 | 31 | male | NA | NA | NA | NA |
| 23 | 53 | male | 1 | 1 | 1 | 0 |
| 24 | 50 | male | 0 | 1 | 1 | 0 |
| 25 | 39 | male | 1 | 1 | 1 | 1 |
| 26 | 48 | male | 1 | 1 | 1 | 0 |
| 27 | 36 | female | 1 | 1 | 1 | 0 |
| 28 | 41 | male | 1 | 1 | 1 | 1 |
| 29 | 48 | female | 1 | 1 | 0 | 1 |
| 30 | 48 | female | 1 | 1 | 1 | 1 |
| 31 | 31 | female | 1 | 1 | 1 | 1 |
| 32 | 2 | male | 0 | 1 | 0 | 0 |
| 33 | 38 | male | 1 | 1 | 1 | 0 |
| 34 | 25 | female | 1 | 1 | 1 | 1 |
| 35 | 15 | male | 1 | 1 | 0 | 1 |
| 36 | 30 | male | 0 | 1 | 1 | 1 |
| 37 | 19 | female | 0 | 1 | 1 | 1 |
| 38 | 37 | male | 0 | 1 | 1 | 1 |
| 39 | 67 | male | 1 | 1 | 1 | 1 |
| 40 | 30 | male | 1 | 1 | 1 | 1 |
| 41 | 37 | male | 0 | 1 | 1 | 1 |
| 42 | 54 | male | 0 | 1 | 1 | 1 |
| 43 | 15 | male | 0 | 0 | 1 | 0 |
| 44 | 21 | male | 1 | 1 | 0 | 0 |
| 45 | 43 | male | 0 | 1 | 1 | 0 |
| 46 | 33 | female | 1 | 0 | 0 | 0 |
| 47 | 42 | male | 0 | 1 | 0 | 1 |
| 48 | 14 | female | 1 | 1 | 1 | 0 |
| 49 | 37 | female | 0 | 1 | 1 | 0 |
| 50 | 7 | male | NA | NA | NA | NA |
| 51 | 8 | female | NA | NA | NA | NA |
| 52 | 53 | male | 1 | 0 | 0 | 0 |
| 53 | 23 | male | 1 | 1 | 1 | 1 |
| 54 | 26 | female | 1 | 1 | 1 | 0 |
| 55 | 24 | female | 1 | 0 | 0 | 0 |
| 56 | 11 | male | 1 | 1 | 0 | 1 |
| 57 | 21 | male | 0 | 0 | 0 | 0 |

| **Case No.** | **Duration of symtoms (mos)** | **location** | **segments** | **Site** | **Surgery** | **Radiotherapy** |
| --- | --- | --- | --- | --- | --- | --- |
| 1 | 6 | Lumbar | 3 | IDEM | 0 | 1 |
| 2 | NA | Thoracic | 6 | IDEM | NA | NA |
| 3 | NA | Lumbar | 1 | IDEM | 0 | 1 |
| 4 | 6 | Lumbar | 3 | IDEM | 1 | 1 |
| 5 | NA | NA | NA | IDEM | NA | 1 |
| 6 | NA | NA | NA | IDEM | NA | 1 |
| 7 | NA | NA | NA | NA | NA | 1 |
| 8 | 36 | Cervicothoracic | 7 | IDEM | 1 | 0 |
| 9 | NA | Lumbar | NA | NA | 0 | 1 |
| 10 | NA | Thoracic | NA | NA | 0 | 1 |
| 11 | NA | Cervical | NA | NA | 0 | NA |
| 12 | 2 | Cervical | 3 | intramedullary | 0 | 1 |
| 13 | NA | Cervical | 4 | intramedullary | 0 | NA |
| 14 | 12 | Cervicothoracic | 8 | intramedullary | 0 | NA |
| 15 | NA | Thoracic | 3 | IDEM | 0 | 1 |
| 16 | 5 | Lumbar | 4 | IDEM | 1 | 1 |
| 17 | 3 | Lumbar | 2 | intramedullary | 1 | 1 |
| 18 | NA | Thoracic | 2 | IDEM | 0 | 0 |
| 19 | 2 | Thoracic | 1 | IDEM | 1 | 0 |
| 20 | 4 | multiple | 6 | IDEM | 1 | 1 |
| 21 | NA | Lumbar | NA | IDEM | 0 | 1 |
| 22 | NA | Cervical | 7 | intramedullary | 0 | 1 |
| 23 | 2 | Cervical | 3 | IDEM | 1 | 1 |
| 24 | 0.5 | Thoracic | 2 | IDEM | 1 | 1 |
| 25 | NA | Lumbar | 5 | IDEM | 0 | 0 |
| 26 | NA | Thoracic | 1 | NA | 1 | 1 |
| 27 | 1 | multiple | 1 | IDEM | 0 | 0 |
| 28 | 2 | multiple | 11 | intramedullary | 0 | 1 |
| 29 | 6 | Thoracic | 3 | intramedullary | 0 | 1 |
| 30 | 48 | Cervical | 6 | intramedullary | 1 | NA |
| 31 | 24 | Thoracic | 3 | intramedullary | 1 | 1 |
| 32 | 3 | Cervical | 2 | intramedullary | 1 | NA |
| 33 | 3 | Cervical | 3 | intramedullary | 1 | NA |
| 34 | 2 | Thoracic | 4 | intramedullary | 1 | NA |
| 35 | 6 | Cervicothoracic | 8 | intramedullary | 0 | 0 |
| 36 | 2 | Lumbar | 4 | intramedullary | 0 | NA |
| 37 | 6 | Cervicothoracic | 7 | intramedullary | 1 | 0 |
| 38 | 12 | Cervicothoracic | 9 | intramedullary | 1 | 0 |
| 39 | 6 | Thoracic | 3 | exophytic | 1 | NA |
| 40 | 6 | Lumbar | 4 | intramedullary | 1 | 1 |
| 41 | 6 | Thoracic | 4 | exophytic | 1 | 1 |
| 42 | 24 | Cervical | 5 | intramedullary | 1 | 1 |
| 43 | 1 | Cervical | 3 | intramedullary | 1 | 1 |
| 44 | 2 | Cervicothoracic | 5 | intramedullary | 1 | 1 |
| 45 | 12 | Cervical | 4 | intramedullary | 1 | 0 |
| 46 | 0.7 | Thoracic | 3 | IDEM | 1 | 1 |
| 47 | 6 | Thoracic | 4 | intramedullary | 1 | 0 |
| 48 | 0.7 | Cervical | 2 | intramedullary | 1 | 1 |
| 49 | 16 | Cervical | 4 | intramedullary | 0 | 1 |
| 50 | NA | Lumbar | 4 | exophytic | 1 | 0 |
| 51 | NA | Lumbar | 2 | exophytic | 0 | 1 |
| 52 | 0.25 | Lumbar | 1 | IDEM | 1 | NA |
| 53 | 2 | multiple | 3 | IDEM | 1 | 1 |
| 54 | NA | multiple | 6 | IDEM | 1 | 1 |
| 55 | NA | multiple | 2 | IDEM | 1 | 1 |
| 56 | 4 | Lumbar | 3 | IDEM | 1 | NA |
| 57 | NA | Lumbar | 5 | IDEM | 0 | 1 |

| **Case No.** | **Chemotherapy** | **PFS1 (mos)** | **Outcome1** | **Progression times** | **OS (mos)** | **Outcome2** |
| --- | --- | --- | --- | --- | --- | --- |
| 1 | 0 | 24 | 1 | 1 | 72 | 1 |
| 2 | NA | NA | NA | NA | NA | NA |
| 3 | 0 | 44.5 | 1 | 1 | 52.5 | 1 |
| 4 | 0 | 34 | 1 | 3 | 95 | 1 |
| 5 | 0 | 200.4 | 1 | 1 | 236.4 | 1 |
| 6 | 0 | 16.8 | 1 | 1 | 19.8 | 1 |
| 7 | 0 | 13.2 | 1 | 1 | 19.2 | 1 |
| 8 | 0 | 6 | 0 | 0 | 6 | 0 |
| 9 | 0 | 12 | 1 | 1 | 18 | 1 |
| 10 | 0 | 36 | 1 | 1 | 54 | 1 |
| 11 | NA | 67 | 0 | 0 | 67 | 0 |
| 12 | 0 | 49 | 1 | 1 | 49 | 0 |
| 13 | NA | NA | NA | NA | NA | NA |
| 14 | NA | NA | NA | NA | NA | NA |
| 15 | 0 | 4 | 1 | 3 | 27 | 1 |
| 16 | 0 | 6 | 1 | 1 | 6 | 1 |
| 17 | 0 | 22 | 1 | 1 | 23 | 0 |
| 18 | 0 | 9 | 1 | 2 | 34 | 1 |
| 19 | 0 | 12 | 1 | 1 | 13 | 1 |
| 20 | 0 | 24 | 1 | 1 | 24 | 0 |
| 21 | 0 | NA | 1 | 1 | 65 | 1 |
| 22 | 0 | 4 | 1 | 2 | 12 | 1 |
| 23 | 0 | 2 | 0 | 0 | 2 | 0 |
| 24 | 0 | 6 | 0 | 0 | 6 | 0 |
| 25 | 0 | 1 | 1 | 3 | 24 | 0 |
| 26 | 0 | 6 | 1 | 3 | 36 | 0 |
| 27 | 0 | 1.633 | 0 | 0 | 1.633 | 1 |
| 28 | 1 | 8 | 0 | 0 | 8 | 0 |
| 29 | 0 | 14 | 1 | 1 | 14 | 0 |
| 30 | NA | NA | NA | NA | NA | NA |
| 31 | 1 | 129 | 0 | 0 | 129 | 0 |
| 32 | NA | NA | NA | NA | NA | NA |
| 33 | NA | 128 | 0 | 0 | 128 | 0 |
| 34 | NA | NA | NA | NA | NA | NA |
| 35 | 0 | NA | 1 | 1 | 23 | NA |
| 36 | NA | NA | NA | NA | NA | NA |
| 37 | 0 | 5 | 0 | 0 | 5 | 1 |
| 38 | 0 | 0.2 | 0 | 0 | 0.2 | 0 |
| 39 | NA | NA | NA | NA | NA | NA |
| 40 | 1 | 94 | 0 | 0 | 94 | 0 |
| 41 | 1 | 78 | 0 | 0 | 78 | 0 |
| 42 | 0 | 31 | 1 | 1 | 31 | 1 |
| 43 | 1 | 78 | 0 | 0 | 78 | 0 |
| 44 | 0 | 69 | 0 | 0 | 69 | 0 |
| 45 | 0 | 73 | 0 | 0 | 73 | 0 |
| 46 | 0 | 71 | 0 | 0 | 71 | 0 |
| 47 | 0 | 64 | 0 | 0 | 64 | 0 |
| 48 | 1 | 51 | 0 | 0 | 51 | 0 |
| 49 | 1 | NA | 1 | 1 | 24 | 1 |
| 50 | 0 | 36 | 1 | 5 | 204 | 1 |
| 51 | 0 | 228 | 0 | 0 | 228 | 0 |
| 52 | NA | NA | NA | NA | NA | NA |
| 53 | 0 | 12 | 0 | 0 | 12 | 0 |
| 54 | 1 | 36 | 0 | 0 | 36 | 0 |
| 55 | 0 | 10 | 1 | 2 | 18 | 0 |
| 56 | NA | NA | NA | NA | NA | NA |
| 57 | 0 | 14 | 1 | 1 | 14 | 0 |

**ESM_3** The Data Extracted from The Included Literature Articles.

For symptoms and treatments, 1 represented “present” and 0 for “absent”. Specially, for surgery, 1 meant gross total resection, while 0 meant otherwise. Outcome1 represented the statuses of tumor progression, while outcome2 represented the statuses of mortality; 1 meant “the occurrence of the event”, while 0 meant “censored”.

*IDEM, intradural extramedullary; OS, overall survival; PFS, progression-free survival.*
