# Supplementary material for: Primary spinal anaplastic ependymoma: A single-institute retrospective cohort and systematic review
Source: Front Oncol. 2023 Feb 7;13:1083085. doi: 10.3389/fonc.2023.1083085 (PMC9941548; doi:10.3389/fonc.2023.1083085)
Supplement: Supplementary file 4 [file Table_4.docx]

| **Supplement Material 4. The Joanna-Briggs Institute Critical Appraisal Tool for Case Reports** | | | | | | | | | |
| --- | --- | --- | --- | --- | --- | --- | --- | --- | --- |
| Publication Year, First author | Were patient’s demographic characteristics clearly described? | Was the patient’s history clearly described and presented as a timeline? | Was the current clinical condition of the patient on presentation clearly described? | Were diagnostic tests or assessment methods and the results clearly described? | Was the intervention(s) or treatment procedure(s) clearly described? | Was the post-intervention clinical condition clearly described? | Were adverse events (harms) or unanticipated events identified and described? | Does the case report provide takeaway lessons? | Points |
| 1980, S.J. MORK | **+** | **+** | **+** | **+** | **+** | **+** | **+** | **+** | **8/8** |
| 1981, Oliver B. | **+** | **-** | **-** | **-** | **-** | **-** | **-** | **+** | **2/8** |
| 1986, Edward G. Shaw | **+** | **-** | **-** | **+** | **+** | **-** | **-** | **+** | **4/8** |
| 1990, Kaoru Fujiyama | **+** | **+** | **+** | **+** | **+** | **+** | **+** | **+** | **8/8** |
| 1995, Shinsuke Katoh | **+** | **+** | **+** | **+** | **+** | **+** | **-** | **+** | **7/8** |
| 2004, N. Fakhrai | **+** | **+** | **+** | **+** | **+** | **+** | **+** | **+** | **8/8** |
| 2005, Raj Kumar | **+** | **+** | **+** | **+** | **+** | **+** | **+** | **+** | **8/8** |
| 2005, Yi-Hsien Lin | **+** | **-** | **-** | **+** | **+** | **+** | **-** | **+** | **5/8** |
| 2006, Alfonso Cerase | **+** | **+** | **+** | **+** | **+** | **-** | **-** | **+** | **6/8** |
| 2006, Mascha Schuurmans | **+** | **+** | **+** | **+** | **+** | **+** | **+** | **+** | **8/8** |
| 2010, Martin Benesch | **+** | **-** | **-** | **+** | **+** | **-** | **-** | **+** | **4/8** |
| 2010, Wook-Ha Kim | **+** | **-** | **-** | **+** | **+** | **-** | **-** | **+** | **4/8** |
| 2011, Michael J. Kinsman | **+** | **+** | **+** | **+** | **+** | **+** | **-** | **+** | **7/8** |
| 2011, Kern H. Guppy | **+** | **+** | **+** | **+** | **+** | **+** | **+** | **+** | **8/8** |
| 2012, Veronique Lorgis | **+** | **+** | **+** | **-** | **+** | **+** | **+** | **+** | **7/8** |
| 2013, Jordi Pe´rez-Bovet | **+** | **+** | **+** | **+** | **+** | **+** | **+** | **+** | **8/8** |
| 2013, Nicolas Nicastro | **+** | **+** | **-** | **+** | **+** | **-** | **-** | **+** | **5/8** |
| 2013, Byung Soo Kim | **+** | **+** | **+** | **+** | **+** | **+** | **+** | **+** | **8/8** |
| 2015, Ihsan Yuce | **+** | **+** | **-** | **-** | **-** | **-** | **-** | **+** | **3/8** |
| 2016, I.J. Pomeraniec | **+** | **+** | **+** | **+** | **+** | **+** | **+** | **+** | **8/8** |
| 2017, Akira Honda | **+** | **+** | **+** | **+** | **+** | **+** | **-** | **+** | **7/8** |
| 2018, Tridu R. Huynh | **+** | **+** | **+** | **+** | **+** | **+** | **+** | **+** | **8/8** |
| 2019, Fabian Montoya | **+** | **+** | **+** | **+** | **+** | **-** | **-** | **-** | **6/8** |
| 2020, Lorenzo Gitto | **+** | **+** | **+** | **+** | **+** | **+** | **-** | **+** | **7/8** |
