# Supplementary material for: Primary spinal anaplastic ependymoma: A single-institute retrospective cohort and systematic review
Source: Front Oncol. 2023 Feb 7;13:1083085. doi: 10.3389/fonc.2023.1083085 (PMC9941548; doi:10.3389/fonc.2023.1083085)
Supplement: Supplementary file 5 [file Table_5.docx]

| **Supplement Material 5. The Joanna-Briggs Institute Critical Appraisal Tool for Case Series.** | | | | | | | | | | | |
| --- | --- | --- | --- | --- | --- | --- | --- | --- | --- | --- | --- |
| Publication Date, first author | Were there clear criteria for inclusion in the case series? | Was the condition measured in a standard, reliable way for all participants included in the case series? | Were valid methods used for identification of the condition for all participants included in the case series? | Did the case series have consecutive inclusion of participants? | Did the case series have complete inclusion of participants? | Was there clear reporting of the demographics of the participants in the study? | Was there clear reporting of clinical information of the participants? | Were the outcomes or follow up results of cases clearly reported? | Was there clear reporting of the presenting site(s)/clinic(s) demographic information? | Was statistical analysis appropriate? | Points |
| 1993, John N. Waldron | **-** | **+** | **-** | **+** | **+** | **+** | **-** | **+** | **+** | **+** | **7/10** |
| 1998, M. Lonjon | **-** | **+** | **+** | **+** | **+** | **+** | **+** | **+** | **+** | **+** | **9/10** |
| 1999, Minoru Hoshimaru | **-** | **+** | **+** | **+** | **-** | **+** | **+** | **+** | **+** | **NA** | **7/9** |
| 2003, Takashi Yokota | **-** | **-** | **-** | **-** | **-** | **+** | **-** | **-** | **+** | **NA** | **2/9** |
| 2013, XiaoDong Liu | **+** | **+** | **+** | **+** | **+** | **+** | **+** | **-** | **+** | **NA** | **8/9** |
| 2014, Tryggve Lundar | **+** | **+** | **+** | **+** | **+** | **+** | **-** | **+** | **+** | **NA** | **8/9** |
